# Digitale Gesundheitsanwendungen (DiGA) als innovativer Baustein in der digitalen Gesundheitsversorgung in Deutschland – Informationen, Erfahrungen und Perspektiven

**DOI:** 10.1007/s00103-021-03420-y

**Published:** 2021-10-05

**Authors:** Wolfgang Lauer, Wiebke Löbker, Thomas Sudhop, Karl Broich

**Affiliations:** 1 Abteilung Medizinprodukte, Bundesinstitut für Arzneimittel und Medizinprodukte (BfArM), Kurt-Georg-Kiesinger-Allee 3, 53175 Bonn, Deutschland; 2 Stabsstelle Innovationsbüro, Changemanagement, Bundesinstitut für Arzneimittel und Medizinprodukte (BfArM), Kurt-Georg-Kiesinger-Allee 3, 53175 Bonn, Deutschland; 3 Abteilung Informationstechnik, Klinische Prüfung, Bundesinstitut für Arzneimittel und Medizinprodukte (BfArM), Kurt-Georg-Kiesinger-Allee 3, 53175 Bonn, Deutschland; 4 Präsident, Bundesinstitut für Arzneimittel und Medizinprodukte (BfArM), Kurt-Georg-Kiesinger-Allee 3, 53175 Bonn, Deutschland

Sehr geehrte Leserinnen und Leser,

E‑Health, Digitalisierung, Gesundheits-Apps – diese Schlagworte sind aktuell in aller Munde. Sie versprechen faszinierende Möglichkeiten der Nutzung innovativer digitaler Technologien und Lösungsansätze in der Gesundheitsversorgung. Gleichzeitig haben sie ein enormes Marktpotenzial mit unmittelbarem Zugang medizinischer Anwendungen zu allen und für alle Nutzerinnen und Nutzer mobiler digitaler Geräte. Damit setzen solche Anwendungen einen Meilenstein in der Gesundheitsversorgung. Zugleich werfen sie regulatorische Fragen auf, z. B. zur Verlässlichkeit der medizinischen Informationen, zum Nachweis des therapeutischen Nutzens sowie zum Datenschutz.

Um gleichzeitig die Chancen innovativer digitaler Ansätze für die medizinische Versorgung in Deutschland zu nutzen und für Patienten wie auch Leistungserbringer Transparenz, Sicherheit und Verlässlichkeit zu schaffen, hat der Gesetzgeber das „Digitale-Versorgung-Gesetz“ (DVG) geschaffen, das im Dezember 2019 in Kraft getreten ist. Auf Basis der dortigen Regelungen in §§ 33a und 139e Fünftes Buch Sozialgesetzbuch (SGB V) haben seither rund 73 Mio. Versicherte in der gesetzlichen Krankenversicherung einen Anspruch auf eine Versorgung mit sogenannten digitalen Gesundheitsanwendungen (DiGA), die von Ärzten und Psychotherapeuten verordnet werden können und durch die Krankenkasse erstattet werden: „Apps auf Rezept“, wie das Bundesministerium für Gesundheit (BMG) diese Neuerung betitelte.

Damit Patientinnen und Patienten gute und sichere innovative Apps etc. schnell nutzen können, wurde für DiGA ein neuer Weg für die Erstattung etabliert: der DiGA-Fast-Track. Dabei spielt das Bundesinstitut für Arzneimittel und Medizinprodukte (BfArM) eine Schlüsselrolle, denn es prüft innerhalb von maximal 3 Monaten nach Antragstellung des Herstellers Sicherheit, Funktionalität, Interoperabilität, Qualität, Datenschutz und -sicherheit der Produkte. Zudem muss der Hersteller zeigen, dass seine App oder Webanwendung einen Beitrag zur Verbesserung der Versorgung in Form „positiver Versorgungseffekte“ (pVE) zeigt, z. B. als Beitrag zur Verbesserung von Krankheitssymptomen, zur Unterstützung der Diagnosefindung oder der Adhärenz zur vereinbarten Therapie einer chronischen Erkrankung. Auch die hierfür vorgelegten Evidenznachweise werden vom BfArM eingehend geprüft. Mit diesem innovativen Verfahren zur Etablierung digitaler Gesundheitsanwendungen als Regelleistung in der gesetzlichen Krankenversicherung ist Deutschland international Vorreiter und viele Länder, vor allem aber unsere europäischen Nachbarn, verfolgen diese Initiative mit großem Interesse.

Das vorliegende Schwerpunktheft „Digitale Gesundheitsanwendungen (DiGA)“ beleuchtet dieses Thema und das konkrete Bewertungsverfahren des BfArM aus unterschiedlichen Perspektiven: patientenbezogen, regulatorisch, wissenschaftlich sowie mit den praktischen Erfahrungen aus dem Verfahren und einem Ausblick auf mögliche zukünftige Entwicklungen rund um die digitale Gesundheitsversorgung.

Der erste thematische Block adressiert die wegbereitenden gesetzlichen Initiativen als Teil der Digitalisierungsstrategie des Bundesministeriums für Gesundheit und die Sicht zentraler Beteiligter des Gesundheitssystems auf Chancen und Risiken.

So ordnen *Ludewig et al.* im ersten Beitrag aus der Perspektive des Bundesministeriums für Gesundheit die neue Möglichkeit zur Einführung digitaler Gesundheitsanwendungen als Regelleistungen der gesetzlichen Krankenversicherung in die digitale Roadmap der Gesundheitsversorgung in Deutschland ein und erläutern das diesbezügliche Ziel der gesetzlichen Regelungen: den schnellen Zugang und Transparenz zu sicheren und mehrwertbringenden digitalen Angeboten.

Patienten und Anwender stehen bei DiGA ganz klar im Zentrum; der nachfolgende Beitrag des „Melanoma Patient Network Europe“ (MPNE) von *Ryll* beleuchtet daher die Patientensicht auf digitale Gesundheitsanwendungen, ihren potenziellen Nutzen und den Bedarf zur Unterstützung, aber auch u. a. zu verlässlichen Qualitäts- und Teststandards sowie der effektiven Integration digitaler Angebote in das Gesundheitswesen und die Patientenversorgung.

Im dritten Beitrag adressieren *Gerlinger et al.* aus Sicht der Leistungserbringer die Voraussetzungen für eine erfolgreiche Integration der DiGA in die Gesundheitsversorgung, insbesondere die Stärkung der Akzeptanz durch erweiterte Daten und Nachweise sowie mehr Transparenz und Erkenntnisse aus der Versorgungsforschung für Verordnende.

*Gregor-Haack et al.* stellen in ihrem Beitrag aus Sicht des GKV-Spitzenverbandes den erforderlichen Weiterentwicklungsbedarf nach den ersten Monaten des neuen Verfahrens dar und nehmen dabei insbesondere den Nutzennachweis und die aktuellen Verfahren zur Preisbildung in den Fokus.

Eine parallele Betrachtung der DiGA-Hersteller mit ersten Erfahrungen und Wünschen an eine Weiterentwicklung u. a. der gesetzlichen Rahmenbedingungen für digitale Anwendungen erfolgt im fünften Beitrag von *Geier* aus Sicht des Spitzenverbandes Digitale Gesundheitsversorgung e. V. (SVDGV).

Im zweiten inhaltlichen Block werden zunächst in einem Beitrag des BfArM (*Lauer et al*.) ausführlich die Vorgehensweise, Prüfkriterien, Ergebnisse und bisherigen Erfahrungen zum „DiGA-Fast-Track“, dem Bewertungsverfahren des BfArM für digitale Gesundheitsanwendungen dargestellt.

Ein weiterer Beitrag von Autorinnen und Autoren des BfArM (*Löbker et al.*) erläutert dessen umfangreiche Informations- und Beratungsangebote rund um DiGA und das Bewertungsverfahren und gibt einen Überblick über die bisherigen Themen und Erfahrungen aus den Beratungen.

Abschließend berichten *Blum et al.* von den praktischen Erfahrungen, Herausforderungen und Erkenntnissen dreier Hersteller von DiGA, die das Bewertungsverfahren des BfArM erfolgreich durchlaufen haben und nachfolgend bereits Erfahrungen in der Kostenerstattung sammeln konnten.

Der dritte Abschnitt fokussiert auf zentrale Prüfbereiche und Kriterien, die DiGA gemäß den Vorgaben der Digitale-Gesundheitsanwendungen-Verordnung (DiGAV) erfüllen müssen.

Dabei stellen die Anforderungen zu Datenschutz und Informationssicherheit aus Datenschutz-Grundverordnung (DSGVO) und DiGAV DiGA-Hersteller teilweise vor nicht unerhebliche Herausforderungen, wie *Zilch und Tschirsich* in ihrem Beitrag darstellen.

*Weber und Heitmann* beschäftigen sich ergänzend mit dem Thema der Interoperabilität als wichtigem Rad im Getriebe eines digitalen, zukunftsorientierten Gesundheitswesens und damit auch wesentlicher Anforderung an DiGA.

Evidenzbasierte Nutzenbewertung, diesbezügliche neue methodische Ansätze zur Studienkonzeption und -durchführung, digitale Biomarker, pragmatische randomisierte Studien und weitere Ansätze zur wissenschaftlichen Evidenzgenerierung bei digitalen Gesundheitsanwendungen sind Gegenstand einer von *Hemkens* verfassten Übersicht und Diskussion.

Ein wichtiges Erfolgskriterium für die Einbindung digitaler Interventionen in die Gesundheitsversorgung bildet die Adhärenz digitaler Interventionen. In diesem Zusammenhang beleuchten *Kernebeck et al.* das Konzept der Adhärenz bei digitalen Interventionen und diesbezüglicher Methoden und Messgrößen und schließen damit den dritten Block ab.

Die Etablierung innovativer digitaler Gesundheitsanwendungen stellt einen wichtigen Schritt auf dem Weg der gesundheitsbezogenen Digitalisierung dar. Zugleich ist sie ein Baustein neben anderen aktuellen spannenden Entwicklungen in diesem Bereich, die zukünftig den Umgang mit unserer Gesundheit und unsere Gesundheitsversorgung prägen werden.

Entsprechend wagen *Brönneke et al.* einen Blick in die Zukunft und auf mögliche, sich bereits heute andeutende Versorgungsszenarien im Jahr 2030 mit ihren Auswirkungen z. B. auf digital integrierte Behandlungspfade weit über eine punktuelle Behandlung im Krankheitsfall hinaus.

Auch das BfArM unterstützt seit vielen Jahren proaktiv die zukunftsorientierte Versorgung von Patentinnen und Patienten mit innovativen Arzneimitteln und Medizinprodukten und gibt in seinem abschließenden Beitrag (*Broich et al.*) einen Überblick zu seinen Initiativen für die Potenzialentfaltung der Digitalisierung, der Digital Readiness, im Gesundheitswesen in Deutschland und Europa.

Liebe Leserinnen und Leser, wir als Editoren dieses Schwerpunktheftes freuen uns, dass die verschiedenen Beiträge einen breiten Überblick über ganz unterschiedliche Aspekte des hochaktuellen und sehr dynamischen Themas digitaler Gesundheitsanwendungen und ihrer noch jungen Etablierung als Regelleistungen der gesetzlichen Krankenversicherung geben. Zugleich regen sie mit konkreten Vorschlägen zur inhaltlichen wie politischen Diskussion im Rahmen der kontinuierlichen Weiterentwicklung des Verfahrens an. Ihnen wünschen wir daher eine interessante und anregende Lektüre.

Dr. Wolfgang Lauer, Dr. Wiebke Löbker, PD Dr. Thomas Sudhop und Prof. Dr. Karl Broich

